# Benign Idiopathic Myoclonus: A New Clinical Entity?

**DOI:** 10.1002/mdc3.70039

**Published:** 2025-03-15

**Authors:** Giorgia Sciacca, A.M. Madelein van der Stouwe, Sterre van der Veen, Hendriekje Eggink, Martje E. van Egmond, Jan Willem J. Elting, Marina A.J. Tijssen

**Affiliations:** ^1^ Expertise Center Movement Disorders Groningen University Medical Center Groningen (UMCG) Groningen The Netherlands; ^2^ Department of Medical, Surgical Sciences and Advanced Technologies GF Ingrassia University of Catania Catania Italy

**Keywords:** hyperkinetic movement disorders, involuntary movements, myoclonus, physiologic myoclonus, physiologic conditions, neurophysiology

## Abstract

**Background:**

Myoclonus is a brief shock‐like, involuntary movement, which can be distinguished in physiologic, essential, epileptic, and symptomatic, according to its etiology. Physiologic myoclonus typically occurs in healthy people without disability or progression.

**Objectives:**

We suggest a new nosological entity in the physiologic group: “benign idiopathic myoclonus.”

**Methods:**

We present a cohort of patients with isolated adolescent‐onset, distal limb myoclonus at rest and during action, in absence of a known cause and disabling progression, who underwent both clinical and neurophysiological examination in our tertiary Movement Disorders Expertise Center Groningen.

**Results:**

Fifteen patients (4 men [26.7%]; age at onset, 18.1 ± 3.6 years; disease‐duration, 5.3 ± 3.7 years) were assessed. Neurophysiological examinations, including electromyography (EMG) (n = 14), somatosensory evoked potentials (SEPs, n = 4); electroencephalography (EEG)–EMG with back‐averaging (BA, n = 11) and cortico‐muscular coherence (CMC, n = 10), confirmed the clinical diagnosis of myoclonus in all patients. Mean EMG burst duration was 62.6 ± 13.7 ms and a cortical origin of myoclonus was demonstrated in six cases (40%). No genetic causes were found. Follow‐up at 0.5 to 8 years depicted clinically stable conditions in eight patients (61.5%), complete remission in four (30.8%), whereas one patient (7.7%) reported slight progression.

**Conclusions:**

We suggest a new phenotype of physiologic myoclonus, which might be called “benign idiopathic myoclonus.” It is characterized by distal myoclonus with onset during adolescence and benign course, without requiring treatment. Clinically and neurophysiologically these jerky movements were compatible with cortical myoclonus in some patients. We were unable to establish any genetic causes in explored cases. This phenotype might represent a particular subgroup of physiologic myoclonus, to be substantiated in multicenter cohorts.

Myoclonus is a hyperkinetic movement disorder, consisting of brief jerks produced by abnormal neuromuscular activation.^1^ It is clinically defined as a sudden, brief, shock‐like, involuntary movement caused by a muscular contraction or inhibition.^2^ A new consensus‐based classification for myoclonus that integrates previous classifications and considers clinical features, anatomical distribution, neurophysiological markers, and etiology has been recently proposed.^3^ The classification is based on two axes: Axis I, which includes a detailed clinical characterization of the patient with myoclonus, and Axis II, which encompasses its etiology.^3^ The inclusion of detailed clinical neurophysiology features in Axis Ib has not been present in previously published classifications, contributing to defining clinical myoclonus syndromes.^3^ Neurophysiological tools, such as multichannel surface electromyography (EMG) recording, electroencephalography (EEG), and EEG–EMG polygraphy with back‐averaging (BA) are indeed useful in characterizing these different forms of myoclonus.^4^

The category of physiologic myoclonus is included in Axis II, according to the new myoclonus classification, which occurs in healthy subjects not associated with disability.^1^, ^2^, ^3^ Normal startle response, hiccups, and myoclonus during sleep are the most familiar examples. The frequency and the severity of physiologic myoclonus are variable. In more exaggerated forms, these features could be the expression of pathological disorders (ie, hyperekplexia), which is featured by an excessive startle response combined with stiffness inducing falls.^5^ Physiological myoclonus can present with a more severe manifestation, with a spontaneous regression, or in the absence of a clinical progression. The clinical presentation of this form of myoclonus might, therefore, be a reason for referral to a movement disorder specialist. Similarly, a high‐frequency myoclonus of the hands might be confused with a tremor syndrome, which also leads to a referral.^6^

Over time, in our tertiary referral center, we identified a specific group of patients with adolescent‐onset of distal upper‐limbs, small amplitude jerks, clinically, and electrophysiologically compatible with myoclonus. A specific genetic etiology was not found and the clinical course was stable or spontaneously improved. These features could be comparable to enhanced physiological tremor (EPT), a well‐recognized tremor entity of physiologically occurring tremor exacerbated by factors such as hyperthyroidism or medication, which is often clinically indistinguishable from small myoclonic jerks in absence of an accurate neurophysiological examination.^6^ Given the lack of a (genetic) etiology and benign disease course in our patients, we consider this identified clinical entity an under‐recognized manifestation of myoclonus that might be called “benign idiopathic myoclonus” (BIM).

We investigated these consecutive patients with clinically relevant myoclonus distally located at the upper extremities, in absence of a specific etiological origin and without disabling features, using standardized clinical and neurophysiological examination. The aim of the study was to systematically describe and further characterize BIM for the first time, as a possible new nosologically entity.

## Methods

### Patient Selection

Patients were retrospectively enrolled at our Movement Disorders Expertise Center, according to the following inclusion criteria: (1) adults with a clinical diagnosis of distal myoclonic jerks, with or without associated head, facial, or perioral jerks; (2) a subjective complaint of mild/moderate jerky movements of the hands; and (3) absence of clinical progression since the onset of the symptoms to the evaluation time. The exclusion criteria were: (1) presence of other neurological signs; (2) occurrence of serum alterations related to metabolic or toxic disorders (renal, hepatic and chronic respiratory failure with hypercapnia, disturbances in glucose homeostasis, hyperthyroidism, and metabolic acidosis or alkalosis); and (3) the use of drugs known to induce myoclonus. All participants were followed up for a minimum of 6 months, ranging to 8 years. Full written informed consent was obtained from all participants according to the Declaration of Helsinki.

### Clinical and Neurophysiological Assessments

At baseline, patients underwent both clinical and neurophysiological examination. Clinical evaluation was conducted by experts in hyperkinetic movement disorders (M.A.J.T., M.E.v.E.). Demographic and clinical characteristics such as gender, age at onset, age at examination, family history, distribution of myoclonus, provoking factors, and stimulus sensitivity were assessed.

The electrophysiological characteristics of myoclonus were investigated as used in our laboratory^7^ by experienced neurophysiologists (J.W.J.E. and M.E.v.E.). Neurophysiological assessment included multichannel surface EMG recording (maximum of 19 channels) with video monitoring, EEG following the 10 to 20 system, EEG–EMG polygraphy with BA, cortico‐muscular coherence (CMC), or somatosensory evoked potentials (SEPs), or a combination of two or more investigations. Surface EMG was recorded from the muscles that appeared to be most affected clinically, and typically included both upper and lower arm flexors and extensors, abductor pollicis brevis, and tibialis anterior in case of leg myoclonus. Spectral analysis using fast Fourier transformation was performed on segments of data in which myoclonus was visible. Comprehensive methods on CMC and BA analysis can be found in the Data S1.

EMG was recorded with Ag/AgCl pairs of surface electrodes placed at affected muscles during rest, posture, application of loading, and action tasks (ie, finger to nose test) evaluating agonist/antagonist burst pattern to differentiate myoclonus (consistently synchronous activation) from tremor (alternating activation),^6^, ^7^, ^8^ burst duration, muscle recruitment, and presence of negative myoclonus. EEG was recorded with Ag/AgCl surface electrodes according to the 10 to 20 International System and acquired by a computerized system (all data was recorded with BrainRT software [OSG BVBA, Rumst, Belgium]) using a sample frequency of 1000 Hz.

If possible, EEG–EMG BA (if >25 jerks) or coherence analysis (if jerk frequency was >1 Hz) were performed. BA was assessed by the presence of a “time‐locked” biphasic potential above baseline on the contralateral motor cortex preceding the EMG registered jerks, with a latency corresponding to the corticomuscular conduction time (ie, +/−20 ms for abductor pollicis brevis, 30 ms for tibialis anterior). The occurrence of significant CMC in the α and β band with a phase difference consistent with a cortical generator was considered indicative of cortical myoclonus. Besides the techniques of BA and CMC, all EEGs were analyzed for epileptiform abnormalities. SEPs were obtained after the stimulation of the mixed median nerve and recorded with Ag/AgCl pairs of surface electrodes on abductor pollicis brevis muscles. Amplitudes of P27 and N35 peaks >5 μV defined the presence of giant SEPs.

## Results

### Clinical Data

Fifteen patients with isolated myoclonic jerks (4 men [26.7%]; age at onset, 18.1 ± 3.6 years; disease duration, 5.3 ± 3.7 years) were enrolled. The demographic and clinical characteristics are summarized in Table 1. All patients did not show any positive family history. Three patients (20%) showed movements phenotypically resembling BIM without additional jerky movements, whereas four of them (26.7%) presented with additional perioral or facial involvement, one with head jerks. Patients 13 and 15, respectively, showed involvement of all limbs and multifocal presentation. A typical example of a patient with BIM is provided in Video 1. Laboratory tests investigating treatable causes of myoclonus (eg, glucose, renal, hepatic, and thyroid function tests) were performed in all patients and additional brain magnetic resonance imaging (MRI) was performed in nine of 15 patients, and all tests came back negative. Nine patients (60%) underwent genetic testing, mostly consisting of genetic panels based on whole exome sequencing (WES) with subsequent analysis of the relevant genes (eg, a dystonia panel [including ANO3, CACNA1B, NKX2‐1/TIF1 and SGCE], myoclonus panel [including ATM, NPC1, and POLG] and/or epilepsy panel [including CACNA1, SCN1A, SLC2A, and STXBP1]), with negative results. Only patient 2 was also tested for intronic repeats with negative results.

**TABLE 1:** Demographic and clinical characteristics

Cases	Gender	Age at onset (y)	Age at time of evaluation (y)	Clinical phenotype	Medication	Neuroimaging	Genetic testing	Follow‐up
No. 1	M	15	21	Distal myoclonus, clinically similar to EPT	Propanolol, no effect	–	–	Stable without medication
No. 2	F	15	22	Distal myoclonus	Valproic acid 300 mg; clonazepam 0.5 mg; levetiracetam 500 mg; propanolol 40 mg; zonisamide 100 mg; no effects	–	Dystonia panel; myoclonus panel; intronic repeats	Slightly progressive, treated with perampanel
No. 3	F	19	21	Subtle distal myoclonus	None	Brain MRI	Myoclonus panel	Stable without medication
No. 4	F	25	28	Perioral and distal myoclonus	Levetiracetam, interrupted for pregnancy	Brain MRI	Myoclonus panel; epilepsy panel	Stable without medication
No. 5	F	17	23	Distal myoclonus	None	–	–	Almost spontaneously disappeared without medication
No. 6	M	21	27	Distal myoclonus, clinically similar to EPT	None	Brain MRI	–	Stable without medication
No. 7	F	14	16	Distal myoclonus	None	Brain MRI	Myoclonus panel	Stable without medication
No. 8	F	17	20	Distal myoclonus	Clonazepam; levetiracetam; propanolol; valproic acid; temporary effects or side effects	Brain MRI	Myoclonus panel	Stable without medication
No. 9	M	16	23	Perioral and distal myoclonus	Levetiracetam 1000 mg; no effect	Brain MRI	Myoclonus panel	Stable without medication
No. 10	M	18	26	Perioral and distal myoclonus	None	Brain MRI	–	No follow‐up
No. 11	F	20	29	Distal myoclonus; slight head jerks	None	–	Myoclonus panel	Stable without medication
No. 12	F	23	26	Distal myoclonus, clinically similar to EPT	Propanolol 80 mg; good effect	Brain MRI	Dystonia panel	Improvement without medication
No. 13	F	17	20	Distal myoclonus in both hands and feet	None	–	–	No follow‐up
No. 14	F	12	27	Facial and distal myoclonus	None	Brain MRI	POLG, MELAS, MERFF, Leigh (elsewhere)	Stable without medication
No. 15	F	22	22	Distal myoclonus, multifocal	Pramipexol; clonazepam 0.5 mg; gabapentin; no effect	–	–	Stable without medication

Abbreviations: M, male; EPT, enhanced physiologic tremor; F, female; MRI, magnetic resonance imaging.

**Video 1:** This is a typical example of a patient with benign idiopathic myoclonus (BIM). The patient is a female adolescent, presenting with distal upper limb myoclonus of relatively small amplitude, in absence of a clinical progression.

At the time of assessment, eight patients (53.3%) had not been previously treated and no medication was prescribed. Two patients reported favorable effects of either propranolol or levetiracetam, the other five patients had tried medication without notable effect (see Table 1).

Clinical follow‐up was possible in 12 patients and depicted globally stable conditions in eight patients (61.5%), complete remission in two (13.3%), whereas one patient (7.7%) reported slight progression. Overall, 12 patients (92.3%) presented with stable conditions or clinical improvement without the need for medication, only one patient was still taking medication (perampanel). Two patients (13.3%) were lost to follow‐up.

### Neurophysiological Data

**FIG. 1:**
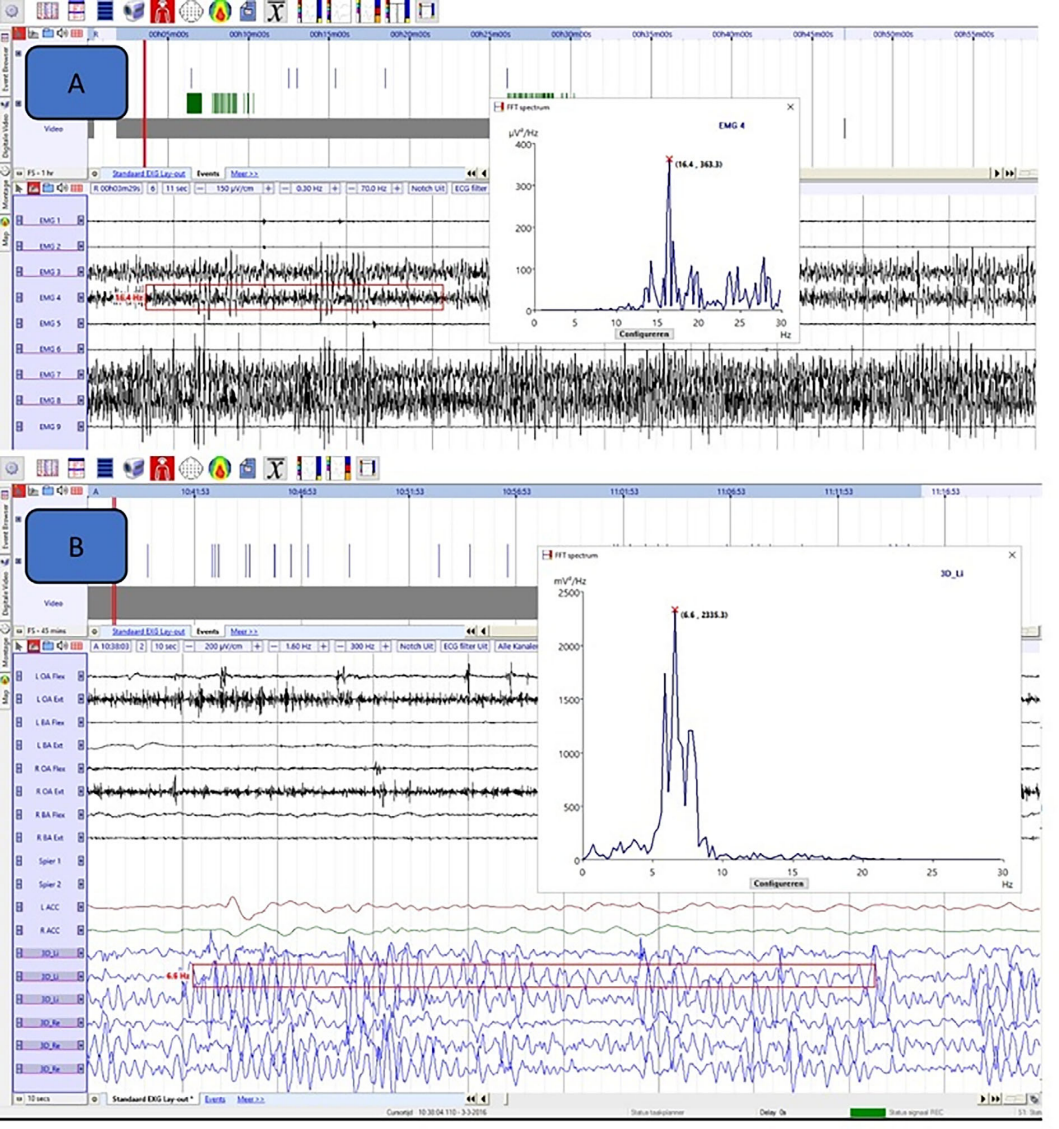
Examples of clinical neurophysiology recordings of two patients, in which Fast Fourier transformations of the electromyography (**A**) patient 4 or accelerometery (**B**) patient 9 signal resulted in a broad peaks in the frequency spectrum, indicating an irregular rhythm. In example (**A**), the dominant frequencies are high (>15 Hz), whereas in example (**B**), the dominant frequencies are in the 5–10 Hz range, whereas both are clearly irregular.

Electrophysiological findings are shown in Table 2. Multichannel surface EMG recording was performed in 14 patients, showing an EMG burst duration of 62.6 ± 13.7 ms with three patients exhibiting a burst duration less than 50 ms (21.4%). The application of loading during EMG polygraphy did not modify duration, amplitude, and frequency parameters in the six patients who underwent the loading test. One of 11 measured patients (9%) showed positive BA. Regarding spectral analysis, we detected no narrow frequency peaks in the power spectra of these patients. If any peak frequency was detected, this consisted of a broad peak indicating an irregular signal, which is consistent with (high frequency) myoclonus.^6^ An example of such a broad frequency peak is provided in Figure 1. Five of 10 patients (50%) had a positive coherence analysis, whereas five (50%) did not show significant peaks on CMC. BA and CMC were not feasible (because of too few jerks or too many artifacts, etc.) or not performed in four (26.7%) and in five (33.3%) patients, respectively. SEPs were performed in four patients and the presence of giant SEPs was observed in one (25%). In particular, patient 4 presented with both giant SEPs and photoparoxysmal response with clear abnormalities at the EEG as additional features.

**Table 2:** Electrophysiological assessment for myoclonus.

Cases	EMG: mean burst duration (ms)	EMG: application of loading	EEG–EMG with BA	CMC	SEPs (μV)
No. 1	73,3 ± 4.9	No effect	Not feasible^a^	Negative	–
No. 2	48,6 ± 10.9	No effect	Negative	Positive at 18–20 Hz	–
No. 3	39,2 ± 6.0	–	Negative	Positive at 19–26 Hz	–
No. 4	–	–	–	Positive at10‐20 Hz; photoparoxysmal response	Giant SEPs: 15 μV
No. 5	72,8 ± 49.8	–	Negative	Negative	No giant SEPs (<3 μV)
No. 6	49,3 ± 4.0	No effect	Not feasible^b^	Not feasible^b^	No giant SEPs (<3 μV)
No. 7	60,9 ± 11.6	No effect	Positive	–	–
No. 8	70 ± 6.8	–	Negative	–	–
No. 9	56,8 ± 5.0	No effect	Negative	Negative	–
No. 10	62 ± 8.2	–	Negative	Positive at 20–26 Hz	–
No. 11	69,3 ± 12.6	No effect	Negative	Not feasible	No giant SEPs (<3 μV)
No. 12	65,4 ± 12.3	–	Negative	Negative	–
No. 13	54,8 ± 5.0	–	–	Positive at 18–22 Hz	–
No. 14	95,4 ± 46.2	–	Negative	Negative	–
No. 15	58,6 ± 3.7	–	Not feasible^b^	–	–

*Note*: Data are reported as means ± standard deviations.

Abbreviations: EMG, electromyography recording; EEG, electroencephalography; BA, EEG–EMG polygraphy with back‐averaging; CMC, cortico‐muscular coherence; SEPs, somatosensory evoked potentials.

^a^ Too few artifacts free burst segments.

^b^ Too few bursts for analysis.

According to both clinical and neurophysiological results, the observed distal jerks were compatible with the diagnosis of myoclonus for all enrolled patients. Cortical origin of myoclonus was definitively demonstrated in six cases (40%).

## Discussion

In the present study, we suggest the existence of a new nosologically myoclonus entity that might be called “benign idiopathic myoclonus.” These jerky movements manifest in the second decade with a predominant distal upper limb distribution and benign course and prognosis. This entity might predominantly affect women who experienced spontaneous improvement or complete remission, and in nearly all cases without requiring pharmacological treatment. The possible etiological causes were ruled out and no triggers were identified.

Physiologic myoclonus is defined as jerky movements that are not related to a pathological condition, but occur in a part of the general population and are considered normal, as with startle,^9^ hiccups, sleep‐onset myoclonus, and myoclonus during syncope^10^, ^11^ and with minimal to no associated disability. BIM could be included in Axis II in this group of physiologic clinical manifestation of myoclonus.

Where the anatomic generator of BIM lies is a challenging question. A short burst duration is suggestive of a cortical origin and recently a threshold of 45 ms was proposed.^3^, ^12^, ^13^, ^14^, ^15^ In our cohort, all patients had an EMG burst duration of less than 100 ms with a mean burst duration of 63 ms (±13.7; range, 39–95 ms). One patient showed a mean burst duration shorter than 45 ms. A cortical correlate was demonstrated by BA or CMC or by the presence of giant SEPs in six patients. Only one of these patients, with a clear cortical origin, showed also an EEG photoparoxysmal response, which required closer follow‐up over time with the aim to observe a possible clinical progression. These findings suggest a cortical myoclonus in some, but not all of our patients, indicating a possibly heterogeneous origin of BIM.

Such heterogeneity with regard to the anatomic generator similarly exists in minipolymyoclonus, which can be either peripheral or cortical in origin. In minipolymyoclonus, the phenomenology is characterized by intermittent, low‐amplitude, arrhythmic movements of the hands (commonly of several fingers) during the maintaining of posture or action.^16^ The pathophysiology of this entity is not clear because it has been described in several disorders such as peripheral nerve hyperexcitability, anterior horn cell disease, but also neurodegenerative disorders such as multiple system atrophy. Neurophysiologically, minipolymyoclonus, is defined as an irregular 1 to 20 Hz activity with muscle synchronous bursts of 25 to 50 ms in duration, deriving from a peripheral or central generator.^17^ In our cohort, the presence of isolated jerky movements with a mean EMG burst duration of 63 ms (±13.7; range, 39–95 ms) and without other neurological manifestation or disease progression, permits to differentiate BIM from minipolymyoclonus.

Myoclonus‐dystonia (MD) presents with a typical onset before 20 years and a fairly benign course, in absence of other severe neurologic deficits. These features are common with BIM. However, in MD, the clinical distribution is typically multifocal and predominately during action with both axial and upper limb involvement, in association to dystonia of a similar distribution. The pathophysiology of MD is subcortical, showing an average EMG duration of 100 ms, with longer duration bursts, whereas no findings of cortical hyperexcitability were found, including a lack of back‐averaged cortical potentials time‐locked to the myoclonus.

In terms of etiology, a wide variety of acquired forms and only a few genetic disorders can manifest as an isolated cortical myoclonus without other signs.^18^ Common metabolic disorders, especially those with a frequent juvenile onset (ie, alterations of glucose homeostasis and hyperthyroidism), could provoke jerky movements.^19^ Moreover, a long list of drugs can cause myoclonus (ie, serotonin reuptake inhibitors, antiepileptic drugs that enhance GABAergic transmission, and tricyclic antidepressants) and their withdrawal could be sufficient for the disappearance of clinical manifestations.^19^ Therefore, it is very important to search for the etiology for adequate and resolutive treatments. All enrolled patients underwent accurate pharmacological anamnesis and specific metabolic screening to rule out these treatable causes. Isolated cortical myoclonus can also rarely have a genetic etiology, such as familial cortical myoclonus tremor and epilepsy (FCMTE) and progressive myoclonus ataxia (PMA).^20^ FCMTE is typically featured by a slowly progressive course, which is less severe compared to other progressive myoclonic epilepsies, with worsening of both tremor and myoclonus.^21^ The phenotype of FCMTE can occasionally be mimicked by Nuclear undecaprenyl pyrophosphate synthase 1 (NUS1) and dehydrodolichol diphosphate synthetase (DHDDS),^22^ however, in most cases, this is accompanied by developmental delay. PMA is defined as a genetic or acquired disorder featured by progressive ataxia and myoclonus, rare epileptic seizures, and without severe cognitive decline.^20^ From our previous study, it appears unlikely that isolated cortical myoclonus could precede PMA, because associated clinical signs, especially ataxia, are often recognized at the beginning of the disease and the age at onset is earlier in PMA (usually approximately 2 years of age) compared to isolated cortical myoclonus.^20^ Our patients did not show any additional clinical sign or progression. In addition, none showed a positive family history and nine of them underwent a comprehensive genetic panel test, which ruled out known genetic disorders. We did not officially rule out intronic repeats for FCMTE in all patients, but the course and lack of family history make that diagnosis very unlikely. Our findings suggest that further genetic investigations are not needed if a patient presents with isolated myoclonic jerks during puberty/adolescence age, in absence of family history, metabolic alterations or relevant medication intake or muscle weakness, which fits the described BIM phenotype. Furthermore, the young age of our cohort of patients suggests that the development of BIM during adolescence period can be related to processes of central nervous system maturation. As observed previously by van Noort and colleagues,^23^ a condition of hyperexcitability can derive from cortical maturation phase, being responsible for a myoclonus of cortical origin. This observation could support the hypothesis of a cortical generator for BIM, as we have hypothesized according to the observed neurophysiological data.

If we recognize BIM as a new entity based on our cohort, this has two practical implications. The first of these relates to diagnostic procedures. If clinicians recognize clinically mild or moderate distal myoclonus, in absence of other neurological signs, especially during the second decade of life, they should consider BIM. In such cases, diagnostic tests could be relatively limited, and routine laboratory tests should be done to rule out treatable causes,^19^ and clinical neurophysiology could be considered to differentiate myoclonus from EPT.^6^ As illustrated by the single patient, an EEG can be of additional value. However, because brain MRI scans and extensive genetic testing yielded no results in our cohort, neuroimaging and genetic tests seem unfeasible, and we would alternatively recommend clinical follow‐up. The second practical implication relates to prognosis and treatment. Because the disease course was favorable in our cohort, with no need for medication at follow‐up in nearly all cases, we recommend that therapeutic management is based only on patients' complaints about the interference of jerky movements on daily activities. Moreover, the possibility of spontaneous improvement or complete remission of complaints should be shared with patients. Of note, these diagnostic and treatment strategies currently do warrant clinical follow‐up, specifically to assess progression of disease severity and the potential development of additional neurological signs and symptoms.

The main limitations of our study are related to the small sample size and to the retrospective design in which patients were specifically selected that fit the predefined phenotype and non‐progressive disease course. We recognize this issue of circularity and see the description of our cohort as a first step in the possible recognition of this myoclonus phenotype. The wide variability in clinical presentation of this new clinical entity, including the presence of slight progression in one case, represents another limit of the study that requires further observations to confirm our hypothesis. Prospective clinical and neurophysiological investigations in a larger population preferably from multiple centers are needed to substantiate our observations. Moreover, future studies are required to determine the exact anatomic origin of BIM and the related pathophysiological mechanisms.

Despite these shortcomings, this new observation may shed light on a newly defined subtype of myoclonus requiring specific and limited diagnostic and therapeutic management.

## Author Roles

(1) Research project: A. Conception, B. Organization, C. Execution; (2) Statistical analysis: A. Design, B. Execution, C. Review and Critique; (3) Manuscript: A. Writing of the First Draft, B. Review and Critique.

G.S.: 1A, 1C, 2A, 2B, 2C, 3A, 3B

A.M.M.v.d.S.: 1A, 1B, 1C, 2C, 3B

S.v.d.V.: 1B, 1C, 3B

H.E.: 1B, 1C, 3B

M.E.v.E.: 1B, 1C, 3B

J.W.J.E.: 1B, 1C, 3B

M.A.J.T.: 1A, 2C, 3B

## Disclosure

**Ethical Compliance Statement**: The study protocol was approved by the ethics committee of the University Medical Centre of Groningen. Full written informed consent was obtained from all participants according to the Declaration of Helsinki. The authors confirm to have read the Journal's position on issues involved in ethical publication and affirm that this work is consistent with those guidelines.

**Funding Sources and Conflict of Interest**: No specific funding was received for this work. The authors declare that there are no conflicts of interest relevant to this work.

**Financial Disclosures for the previous 12 months**: The authors declare that there are no additional disclosures to report.

## Supporting information


**Data S1.** Supporting Information.

## Data Availability

The data that support the findings of this study are available on request from the corresponding author. The data are not publicly available due to privacy or ethical restrictions.
