# Supplementary material for: Benign Idiopathic Myoclonus: A New Clinical Entity?
Source: Mov Disord Clin Pract. 2025 Mar 15;12(7):938–46. doi: 10.1002/mdc3.70039 (PMC12275006; doi:10.1002/mdc3.70039)
Supplement: Supplementary file 1 — Data S1. Supporting Information. [file MDC3-12-938-s001.docx]

**Supplementary Materials**

**Neurophysiological methods procedures**

- **EEG-EMG Back-averaging procedure:**

1. Select artefact free segments from the EEG/EMG recording, with clearly present myoclonus, on the EMG and visually verified on the video recording.
2. Export these segments to Brain Vison Analyzer.
3. Use a 10 Hz high pass zero phase shift filter on the EMG channel, 48 db/octave.
4. Optionally, use a 1 Hz high passe filter on the EEG channels, in case of slow drift artefacts.
5. Place markers at the onset of myoclonus on the EMG channel.
6. Segment the data using 100 ms before and 50 ms after the onset of myoclonus.
7. Average all available segments.
8. EEG spike identification: this is based on combined anatomical, physiological and statistical plausibility.
   1. Use voltage topographical mapping to assist in EEG voltage field inspection;
   2. EEG pre-myoclonus spike needs to be on the contralateral side to the myoclonus for arm myoclonus (C3 or C4), or midline (Cz) for leg myoclonus. i.e check for topographical/anatomical plausibility;
   3. EEG pre-myoclonus spike needs to have the correct latency with regard to the onset of the myoclonus i.e. +/- 20 ms for APB, 30 ms for tibialis anterior) so corresponding to the cortico-muscular latency that fits the pyramidal tract conduction to that muscle. i.e. check for physiological plausibility;
   4. Should be clearly higher in voltage than the baseline segment that extends from -100 to -50 ms from myoclonus onset. See also step e;
   5. Check the consistency of the result using odd-even averaging, i.e. create different sub-averages based on odd an even data segment. Alternatively, Z-score mapping, i.e. expressing the EEG voltage values as Z-scores deviation from the value 0 (which is the expected value when no pre-myoclonus spike is present) can be used to check if the EEG spike is significantly different from 0. i.e. check for statistical plausibility.

- **Somatosensory evoked potentials (SEPs)** **procedure:**

1. The number of stimuli is between 300-500 stimuli, depending on the noise level.
2. We do sometimes record the CMAP form the APB just to verify the adequacy of the electrical stimulation, but the SEP itself was measured from paracentral electrodes: CP3-CP4 for right arm stimulation; CP4-Fz or CP4-CP3 for left arm stimulation.
3. Baseline to top amplitude is measured from the N20.

- **Cortico-muscular coherence (CMC) analysis:**

1. Select a continuous artefact free segment from the EEG/EMG recording, with clearly present myoclonus, on the EMG and visually verified on the video recording. Frequency of the myoclonus should be at least 1 Hz. Use source derivation for the EEG channels. At least 30 seconds of data should be present.
2. Export these segments to an ASCII text file, at 1024 Hz. Use inhouse written software to perform the coherence analysis.
3. Use a 10 Hz high pass zero phase shift filter on the EMG channel, 48 db/octave.
4. Rectify the EMG channel.
5. Perform coherence analysis between the contralateral EEG electrode (C3, C4, or Cz in case of leg myoclonus) and the EMG channel using 1 second data segments with 50% overlapping windows using a Hanning window.
6. Calculate the ratio of the squared cross spectrum and the product of the autospectra to find the coherence spectrum.
7. Check if significant coherence exists between 10 and 30 Hz, for at least 5 consecutive bins, with significance derived from the formula 1-(0.05)1(/L-1) where L is the number of averages used in the coherence calculation.
8. In case of significant coherence in step 7: check for topographical/anatomical plausibility by verifying that the ipsilateral electrode does not have significant coherence.
9. Check for physiological plausibility by inspecting the phase spectrum and estimate the cortico-muscular conduction time from the group delay phenomenon if present. Linear trends in the phase spectrum should be either descending (indicating cortico-muscular conduction) or flat (indicating a mix of cortico-muscular and musculo-cortical conduction), but not ascending (indicating musculo-cortical conduction only).

**Muscles recorded in each patient**

| **Cases** | **Recorded muscles** |
| --- | --- |
| n. 1 | biceps, triceps, lower arm flexor and extensor muscles, abductor pollicis brevis. |
| n. 2 | biceps, triceps, lower arm flexor and extensor muscles, abductor pollicis brevis, rectus femoris, tibialis anterior, extensor digitorum brevis. |
| n. 3 | biceps, triceps, lower arm flexor and extensor muscles, abductor pollicis brevis. |
| n. 4 | biceps, triceps, lower arm flexor and extensor muscles, abductor pollicis brevis. |
| n. 5 | biceps, triceps, lower arm flexor and extensor muscles. |
| n. 6 | biceps, triceps, lower arm flexor and extensor muscles. |
| n. 7 | biceps, triceps, lower arm flexor and extensor muscles. |
| n. 8 | biceps, triceps, lower arm flexor and extensor muscles, abductor pollicis brevis, abductor minimi digiti. |
| n. 9 | biceps, triceps, lower arm flexor and extensor muscles. |
| n. 10 | biceps, triceps, lower arm flexor and extensor muscles. |
| n. 11 | biceps, triceps, lower arm flexor and extensor muscle, sternocleidomastoid, abductor pollicis brevis. |
| n. 12 | biceps, triceps, lower arm flexor and extensor muscles. |
| n. 13 | biceps, triceps, lower arm flexor and extensor muscle, abductor pollicis brevis, rectus femoris, biceps femoris, tibialis anterior, gastrocnemius. |
| n. 14 | biceps, triceps, lower arm flexor and extensor muscles, abductor pollicis brevis. |
| n. 15 | biceps, triceps, lower arm flexor and extensor muscles, abductor pollicis brevis. |

**Genes per genetic panel**

Panels are based on Whole Exome Sequencing (WES) and subsequent analysis of coding sequencies and flanking intron sequencies of the genes in each panel.

*Myoclonus panel*

ADCK3, ADCY5, AMT, ANO3, ARHGEF9, ASAH1, ATM, ATP13A2, ATP7A, ATP7B, BRAT1, CACNA1A, CACNA1B, CACNB4, CAMTA1, CARS2, CASR, CDKL5, CERS1, CHD2, CLCN2, CLN3, CLN5, CLN6, CLN8, CNTN2, CSTB, CYP27A1, DNAJC5, DNAJC6, EFHC1, EIF2B5, EPM2A, FOLR1, FOXG1, GABRA1, GABRD, GABRG2, GALC, GBA, GCSH, GFAP, GLDC, GLRA1, GLRB, GNAO1, GOSR2, HEXA, HEXB, KCNC1, KCNC3, KCND3, KCTD17, KCTD7, KMT2B, MECP2, MFSD8, NEU1, NHLRC1, NKX2-1, NPC1, NPC2, PANK2, PCDH19, PIGA, POLG, PPT1, PRICKLE1, PRKCG, PSAP, RELN, RNASEH2A, RNASEH2B, RNASEH2C, RPS6KA3, SACS, SAMHD1, SCARB2, SCN1A, SCN2A, SCN8A, SCN9A, SGCE, SLC2A1, SLC6A1, SLC6A5, STXBP1, TBC1D24, TH, TPP1, TREX1, UBE3A

*Dystonia panel*

ADAR, ADCY5, ALDH5A1, ANO3, ARX, ATP13A2, ATP1A3, ATP7B, BCS1L, C10ORF2, C19ORF12, CACNA1B, CDKL5, CIZ1, COX10, COX15, COX20, CP, DDC, DLAT, DLD, FA2H, FBXO7, FOLR1, FOXG1, FTL, FUS, GCDH, GCH1, GNAL, LRPPRC, MECP2, MTTP, NDUFA10, NDUFA12, NDUFA2, NDUFA9, NDUFAF2, NDUFAF5, NDUFAF6, NDUFS1, NDUFS3, NDUFS4, NDUFS7, NDUFS8, NKX2-1, NPC1, NPC2, NUP62, PAH, PANK2, PARK2, PARK7, PCBD1, PDHA1, PDHB, PDHX, PINK1, PLA2G6, PLP1, PNKD, POLG, PRKRA, PRRT2, PTS, QDPR, RNASEH2A, RNASEH2B, RNASEH2C, SAMHD1, SCO2, SERAC1, SGCE, SLC16A2, SLC19A3, SLC20A1, SLC2A1, SLC30A10, SLC6A19, SLC6A3, SPG11, SPG7, SPR, SUCLA2, SUCLG1, SURF1, TACO1, TAF1, TH, THAP1, TIMM8A, TOR1A, TREX1, TUBB4A, VPS13A, WDR4

*Epilepsy panel*

AARS, ADSL, ALDH7A1, ALG13, AMT, AP3B2, ARHGEF9, ARX, ASAH1, ATP1A2, ATP1A3, ATP6AP2, ATRX, CACNA1A, CACNB4, CASK, CASR, CBS, CDKL5, CHD2, CHRNA2, CHRNA4, CHRNB2, CLN3, CLN5, CLN6, CLN8, CNKSR2, CNTNAP2, CPA6, CPT2, CTSD, CUL4B, DCX, DENND5A, DEPDC5, DNAJC5, DNM1, DOCK7, DYRK1A, EEF1A2, EFHC1, EPM2A, FGD1, FLNA, FOLR1, FOXG1, FRRS1L, GABRA1, GABRA3, GABRB2, GABRB3, GABRD, GABRG2, GAMT, GATM, GCSH, GLDC, GLRA1, GLRB, GNAO1, GOSR2, GPC3, GPHN, GRIA3, GRIK2, GRIN1, GRIN2A, GRIN2B, GRIN2D, GRN, HCFC1, HCN1, HDAC4, HNRNPU, HPRT1, HSD17B10, IQSEC2, KCNA2, KCNB1, KCNC1, KCNH1, KCNJ10, KCNMA1, KCNQ2, KCNQ3, KCNT1, KCTD7, KDM5C, KPNA7, LGI1, MBD5, MDH2, MECP2, MED12, MEF2C, MFSD8, MOCS1, MOCS2, MTHFR, NHLRC1, NPRL2, NPRL3, NRXN1, NSDHL, OFD1, OPHN1, PAK3, PCDH19, PHF6, PIGA, PIGN, PIGT, PLCB1, PLP1, PNKP, PNPO, POLG, PPT1, PQBP1, PRICKLE1, PRICKLE2, PRPS1, PRRT2, PSAT1, PSPH, PURA, RAB39B, RAI1, RANBP2, RARS2, RELN, RNASEH2A, RNASEH2B, RNASEH2C, ROGDI, SAMHD1, SCARB2, SCN1A, SCN1B, SCN2A, SCN8A, SIK1, SLC12A5, SLC13A5, SLC16A2, SLC19A3, SLC1A3, SLC25A15, SLC25A22, SLC2A1, SLC35A2, SLC6A1, SLC6A5, SLC6A8, SLC9A6, SMC1A, SMS, SPTAN1, ST3GAL3, ST3GAL5, STX1B, STXBP1, SYN1, SYNGAP1, SYNJ1, SYP, SZT2, TBC1D24, TBCE, TCF4, TPP1, TREX1, UBA5, UBE2A, UBE3A, WDR45, WWOX, YWHAG, ZEB2
